# Death Rate

**Published:** 1873-07

**Authors:** 


### DEATH RATE.

WITHIN the last three hundred years the average duration of human life among civilized nations has nearly doubled. The first attempt to ascertain the average of life was made by the Roman Emperor Severus, about 250 years after Christ. Among the upper classes it was thirty years; that is, taking any ten persons, their united ages at death added up would make 300 years. Taking rich and poor throughout the empire it was under twenty; it is now over forty years in Europe and the United States. The main reason for ascertaining this was to find out the present value of an estate, for example, which would come into the possession of B when A died; the older A was the more it would be worth to B.

In Switzerland, in 1550, the average of life was about twenty-one years; in 1850 it was forty-one years—nearly double. English speaking people have an average length of life about double that of the old Romans; and while the most favored classes among the Romans lived thirty years, the same classes in England and America now average sixty years.

The lowest ascertained death rate in the civilized world was in the islands on the northern coast of Scotland, where twelve persons out of one thousand die annually. The highest was in Paris during 1871, as a result of siege, and fever, and want—being fifty-five per thousand; next to that is Amsterdam, forty-six; and Rotterdam, at the mouth of the Rhine, thirty-four. In sixty-three counties in England and Wales, among a population mostly rural, there is an average from year to year of seventeen deaths among a thousand persons. Taking the whole of England togethei it is twenty-three, of London twenty-two, of the United States twenty-four.

From official sources the mortality of 1871, in a number of prominent cities, is ascertained to be as in the following tables—one alphabetical, the other giving the highest first, for convenience of reference, enabling the reader to decide at once as to the comparative healthfulness of localities. It is customary to estimate that for every death there are twenty-eight days of sickness among the people; that is, where one man dies twenty-eight have been sick one day. That should be considered a very healthy locality where only fifteen persons die out of every thousand in any one year. There are very few such favored places on the globe.

The reader should bear in mind that the lowest death rate, found in modern times, is twelve persons out of one thousand dying every year, taking young and old together. In some years the death rate of New York City has been thirty-six per thousand, while that of the whole country is twenty-six, and of England twenty-four.

For every person that dies twenty-eight are sick, and many of these from preventable causes. Scientific men have estimated that in Great Britain one hundred thousand out of thirty millions die every year unnecessarily, who would not have died if proper care had been taken as to habits of life. The hope is that the time is coming when the intelligence of the people will lead them to take as much care in preserving their health as their money.

| PLACE. | DEATHS PER 1,000 ANNUALLY. | PLACE. | DEATHS PER 1,000 ANNUALLY. |
|---|---|---|---|
| Albany, N. Y | 24 | Louisville, Ky | 24 |
| Amsterdam, Holland | 34 | Manchester, Eng | 31 |
| Baltimore, Md | 27 | Milan | 38 |
| Berlin, Ger | 39 | Mobile | 35 |
| Birmingham, Eng | 25 | Montreal | 37 |
| Boston, Mass | 24 | Naples | 39 |
| Brighton, Eng | 23 | Newark, N. J | 24 |
| Brooklyn, N. Y | 26 | Newcastle, Eng | 32 |
| Brussels, Greece | 32 | New Orleans, La | 30 |
| Buffalo, N. Y | 14 | Paris, France | 55 |
| Charleston, S. C | 31 | Philadelphia, Pa | 23 |
| Chicago, Ill | 23 | Petersburg, Va | 44 |
| Cincinnati, O | 23 | Plymouth, Eng | 34 |
| Cleveland, O | 20 | Richmond, Va | 34 |
| Copenhagen | 26 | Rome, Italy | 34 |
| Detroit | 22 | Rotterdam | 46 |
| Dublin | 26 | St. Louis, Mo | 17 |
| Edinburgh | 27 | Savannah, Ga | 31 |
| Florence | 38 | Salt Lake City | 34 |
| Faroe Islands | 12 | San Francisco | 22 |
| Genoa | 29 | Sheffield, Eng | 28 |
| Glasgow | 33 | Sunderland, Eng | 36 |
| Havana | 46 | Troy, N. Y | 38 |
| Hoboken, N. J | 31 | Turin | 33 |
| Leeds, Eng | 26 | Vienna | 32 |
| Liverpool, Eng | 35 | Vicksburg, Miss | 46 |
| London, Eng | 25 | Washington City | 14 |

